# Effects of the alpine meadow in different phenological periods on rumen fermentation and gastrointestinal tract bacteria community in grazing yak on the Qinghai-Tibetan Plateau

**DOI:** 10.1186/s12866-024-03182-y

**Published:** 2024-02-19

**Authors:** Tongqing Guo, Xungang Wang, Qian Zhang, Yuna Jia, Yalin Wang, Lin Wei, Na Li, Xianli Xu, Hongjin Liu, Linyong Hu, Na Zhao, Shixiao Xu

**Affiliations:** 1 Northwest Institute of Plateau Biology, Chinese Academy of Sciences, Xining, 810008 China; 2 University of Chinese Academy of Sciences, Beijing, 100049 China

**Keywords:** Grazing strategy, Rumen fermentation, Bacteria function prediction, Gastrointestinal microorganisms, Ruminant

## Abstract

**Background:**

In this study, we investigated the effects of alpine meadow in different phenological periods on ruminal fermentation, serum biochemical indices, and gastrointestinal tract microbes in grazing yak on the Qinghai-Tibetan Plateau. A total of eighteen female freely grazing yaks with an average age of 3 years old and a body weight of 130 ± 19 kg were selected. According to the plant phenological periods, yaks were randomly allocated to one of three treatments: (1) regreen periods group (RP, *n* = 6); (2) grassy periods group (GP, *n* = 6); and (3) hay periods group (HP, *n* = 6). At the end of the experiment, the blood, rumen fluids, and rectal contents were collected to perform further analysis.

**Results:**

The concentrations of total volatile fatty acid (TVFA), acetate, glucose (GLU), triglyceride (TG), cholesterol (CHO), high density lipoprotein (HDL), and low density lipoprotein (LDL) were higher in the GP group than in the HP group (*P* < 0.05). However, compared with the RP and GP groups, the HP group had higher concentrations of isobutyrate, isovalerate, valerate, and creatinine (CREA) (*P* < 0.05). The abundance of *Prevotella* in the rumen, and the abundances of *Rikenellaceae_RC9_gut_group*, *Eubacterium_coprostanoligenes_group*, and *Prevotellaceae_UCG-004* in the gut were higher in the GP group compared with the HP group (*P* < 0.05). The HP had higher abundance of *Eubacterium_coprostanoligenes_group* in the rumen as well as the abundances of *Romboutsia* and *Arthrobacter* in the gut compared with the RP and GP groups (*P* < 0.05).

**Conclusions:**

Based on the results of rumen fermentation, serum biochemical, differential biomarkers, and function prediction, the carbohydrate digestion of grazing yak would be higher with the alpine meadow regreen and grassy due to the gastrointestinal tract microbes. However, the risk of microbe disorders and host inflammation in grazing yak were higher with the alpine meadow wither.

**Supplementary Information:**

The online version contains supplementary material available at 10.1186/s12866-024-03182-y.

## Background

The yak (*Bos grunniens*), an indigenous herbivore raised at average altitudes 4000 m above sea level. It plays a vital role in the culture and livelihoods such as ecological stability, socio-economic development [1]. In this region, the yaks endure an extremely harsh conditions, including low air temperatures and oxygen content, high UV light and winds, and severe snowstorms. Ruminants themselves do not produce the enzymes needed to breakdown the most complex plant polysaccharides, which relay on the rumen microbiota [2]. Moreover, the rumen bacteria of yak are associated with feed efficiency, which is also subject to interaction with its host as part of co-evolutionary responses to challenge natural environments [3]. It seems that the microbes in yak appear to be highly sensitivity to seasonal variability [4].

In tradition, yaks graze the grasslands throughout year without any extra supplemental feed. Alpine meadow turns green in May, maintains a grassy green from June to October, and withers in November. A brief period of growth ranging from 90 to 120 days results in a fluctuating supply of alpine meadow during the long, cold winter. In addition, the biomass was higher in August at 286.5 g/m^2^ than in the December (121.4 g/m^2^). The crude protein (CP) in grass was higher in the regreen stage at 12.8% of dry matter (DM), but the CP content in grass was lower in the hay stage at 6.7%. On the contrary, the NDF and ADF contents were higher in the hay stage at 62.3% and 36.3% of DM, respectively [5]. As a result, the nutrient composition of alpine meadow fluctuates greatly, resulting in not getting maintenance requirements for grazing yaks in winter. It has been speculated that the dynamics of alpine meadow affect the composition of rumen bacteria. A previous study on the yak reported the effect of seasonal changes in nutrient availability on the rumen microbiome by simulating grazing conditions in winter pasture and warm season pasture [6]. However, these studies did not consider the dynamics of nutrients with the alpine meadow growth in a special geographical environment. The effect of the alpine meadow in different phenological periods on the gastrointestinal tract microbes of the yak remain unclear.

The objective of this study was to determine the effects of alpine meadow in different phenological periods on the gastrointestinal tract microbes in grazing yak. We hypothesized that the alpine meadow in different phenological periods influenced the bacteria composition of gastrointestinal tract, improving the adaptation to the plateau. These results will be important in understanding the role of microbes in grazing yak with alpine meadow growth.

## Results

### Characteristics of rumen fermentation

As shown in Table 1, the total concentration of VFA in the RP and GP groups was higher than in the HP group (*P* = 0.006). Compared to the RP and HP groups, the proportion of acetate was greater in the GP group (*P* = 0.014). However, the proportions of isobutyrate, isovalerate, and valerate were higher in the HP group than those of the RP and GP groups (*P* < 0.05). The RP group had greater proportion of butyrate compared to the GP and HP groups (*P* < 0.001). No difference in the proportion of propionate and the ratio of acetate to propionate was detected among the three groups (*P* > 0.05).

**Table 1:** Effects of the alpine meadow in different phenological periods on the rumen fermentation paraments

	RP	GP	HP	SEM^1^	P-value
TVFA^2^, mmol/L	100.97^a^	122.39^a^	62.09^b^	8.924	0.006
Acetate, %	71.15^b^	74.96^a^	71.54^b^	0.639	0.014
Propionate, %	12.93	13.50	13.49	0.187	0.403
Isobutyrate, %	1.61^b^	1.48^b^	2.16^a^	0.121	0.031
Butyrate, %	11.18^a^	6.70^b^	7.80^b^	0.519	< 0.001
Isovalerate, %	2.06^b^	2.44^b^	3.78^a^	0.272	0.014
Valerate, %	1.07^b^	0.92^b^	1.23^a^	0.056	0.062
A:P^3^	5.52	5.57	5.32	0.086	0.473

^1^SEM, standard error of the mean; ^2^TVFA, total of volatile fatty acids; ^3^A:P, the ratio of acetate to propionate. a − c means within a row with different subscripts differ when *p*-value < 0.05

### Serum biochemical indices

As shown in Table 2, the concentrations of GLU, TG, CHO, HDL, and LDL were higher in the GP group compared to the HP group (*P* < 0.05). The RP group had greater concentrations of TBA, UREA, and blood urea nitrogen (BUN) than those in the GP and HP groups (*P* < 0.05). The concentration of CREA was higher in the HP group compared with the GP and HP groups (*P* < 0.05). No difference in ALT and AST concentration was observed among the three groups (*P* > 0.05).

**Table 2:** Effects of the alpine meadow in different phenology periods on the serum biochemical indices in grazing yaks

	RP	GP	HP	SEM^1^	P-value
ALT^2^, U/L	49.04	38.68	38.14	3.167	0.302
AST^3^, U/L	140.02	87.77	80.69	12.708	0.110
ALB^4^, g/L	39.94^b^	44.88^a^	39.63^b^	1.039	0.058
TBA^5^, µmol/L	24.82^a^	7.72^b^	24.19a^b^	3.004	0.020
UREA^6^, mmol/L	9.04^a^	5.47^b^	2.14^c^	0.726	< 0.001
BUN^7^, mmol/l	25.31^a^	15.30^b^	6.18^c^	2.008	< 0.001
CREA^8^, µmol/L	95.18^c^	166.77^b^	195.46^a^	11.136	< 0.001
GLU^9^, mmol/L	4.10^b^	5.69^a^	3.99^b^	0.318	0.039
TG^10^, mmol/L	0.38^a^	0.43^a^	0.21^b^	0.034	0.010
CHO^11^, mmol/L	3.04^ab^	3.32^a^	2.33^b^	0.173	0.042
HDL^12^, mmol/L	2.22^a^	2.17^a^	1.67^b^	0.094	0.018
LDL^13^, mmol/L	0.65^b^	0.87^a^	0.53^b^	0.050	0.007

^1^SEM, standard error of the mean; ^2^ALT, Glutamic-pyruvic Transaminase; ^3^AST

Glutamic oxaloacetic Transaminase; ^4^ALB, Albumin; ^5^TBA, Total bile acids; ^6^UREA, Urea; ^7^BUN, Blood urea nitrogen; ^8^CREA, Creatinine; ^9^GLU, Glucose; ^10^TG, Triglyceride; ^11^CHO, Cholesterol; ^12^HDL, High density lipoprotein; ^13^LDL, Low density lipoprotein

a − c means within a row with different subscripts differ when p-value < 0.05

### Diversity of the bacterial community in the rumen

**Fig. 1:**
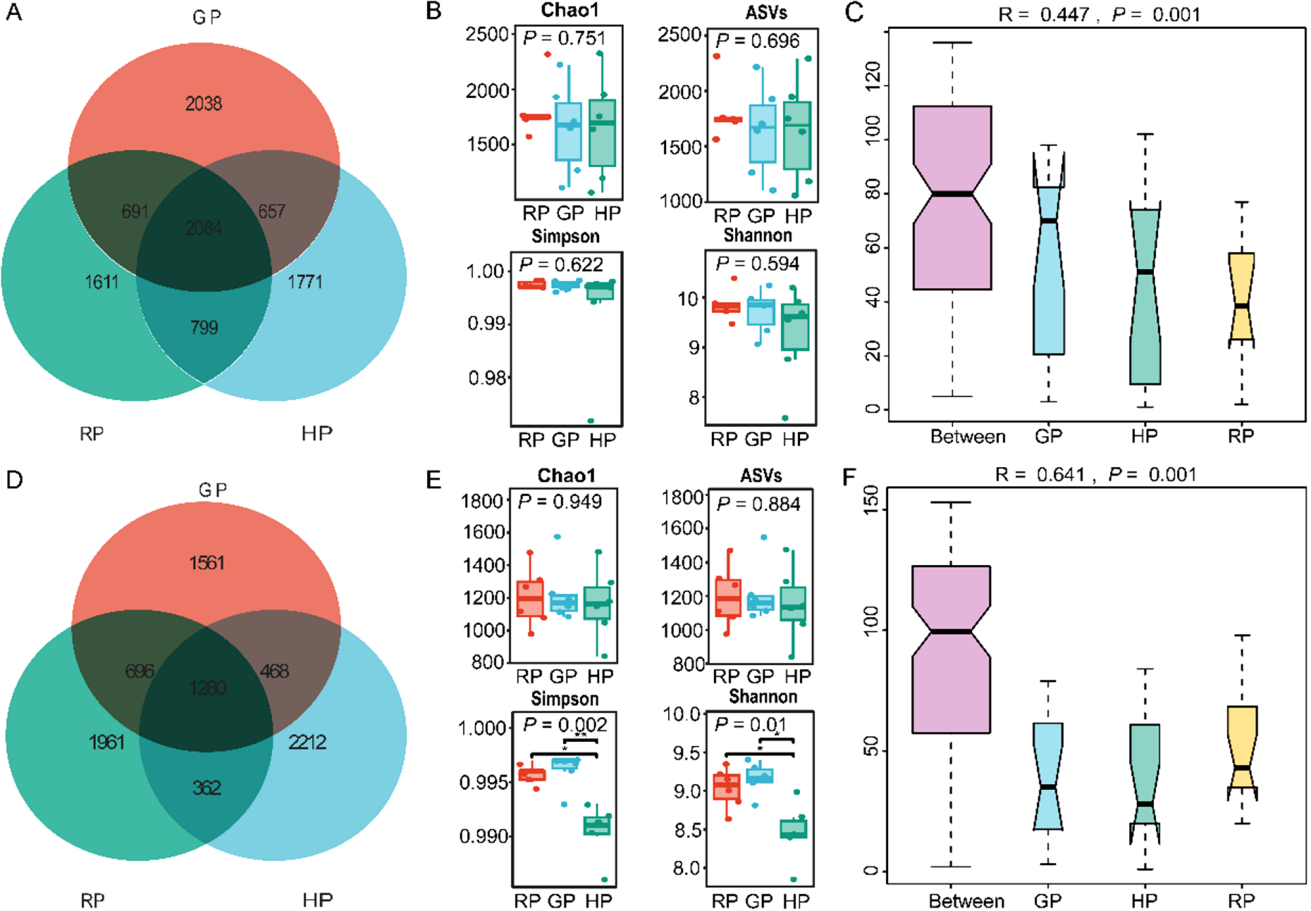
The venn diagram illustrates microbial ASVs overlaps in the rumen between the three groups (**A**). Alpha diversity of the ruminal bacteria between the three groups (**B**). The PERMANOVA test in the ruminal bacterial community based on the bray-curtis dissimilarity (**C**). Venn diagram demonstrated bacterial ASVs in the gut among the experimental treatments (**D**). Alpha diversity of bacterial samples in the gut (**E**). The PERMANOVA test in the gut bacterial community based on the Bray-Curtis distance dissimilarity (**F**)

After the quality filtering step, a total of 9651 ASVs were obtained from 16 S rRNA high-throughput sequencing of three groups (Fig. 1A). Of these, 1611, 2038, and 1771 specific ASVs were observed in the RP, GP, and HP groups, respectively. We calculated 4 estimates of bacteria community alpha diversity (Chao1, ASVs, Simpson, and Shannon) on individual groups (Fig. 1B). No significant difference was found in the alpha diversity indices (*P* > 0.05). The PERMANOVA test based on the Bray-Curtis distance measures showed that significant differences in ruminal bacterial community composition among the three groups (*P* = 0.001) (Fig. 1C).

### Bacterial diversity in the gut

With regard to the gut bacteria community, a total of 8540 ASVs were found from three groups (Fig. 1D). Among them, 1961, 1561, and 2212 specific ASVs were identified in the RP, GP, and HP groups, respectively. Figure 1E depicted the alpha diversity indices in the gut. Apart from the indices of Chao1 and ASVs, the Simpson and Shannon indices were higher in the GP group compared to the RP and HP groups (*P* < 0.01). The PERMANOVA test based on the Bray-Curtis distance measures revealed that significant differences in gut bacterial community composition among the three groups (*P* = 0.001) (Fig. 1F).

### Bacterial composition in the rumen

**Fig. 2:**
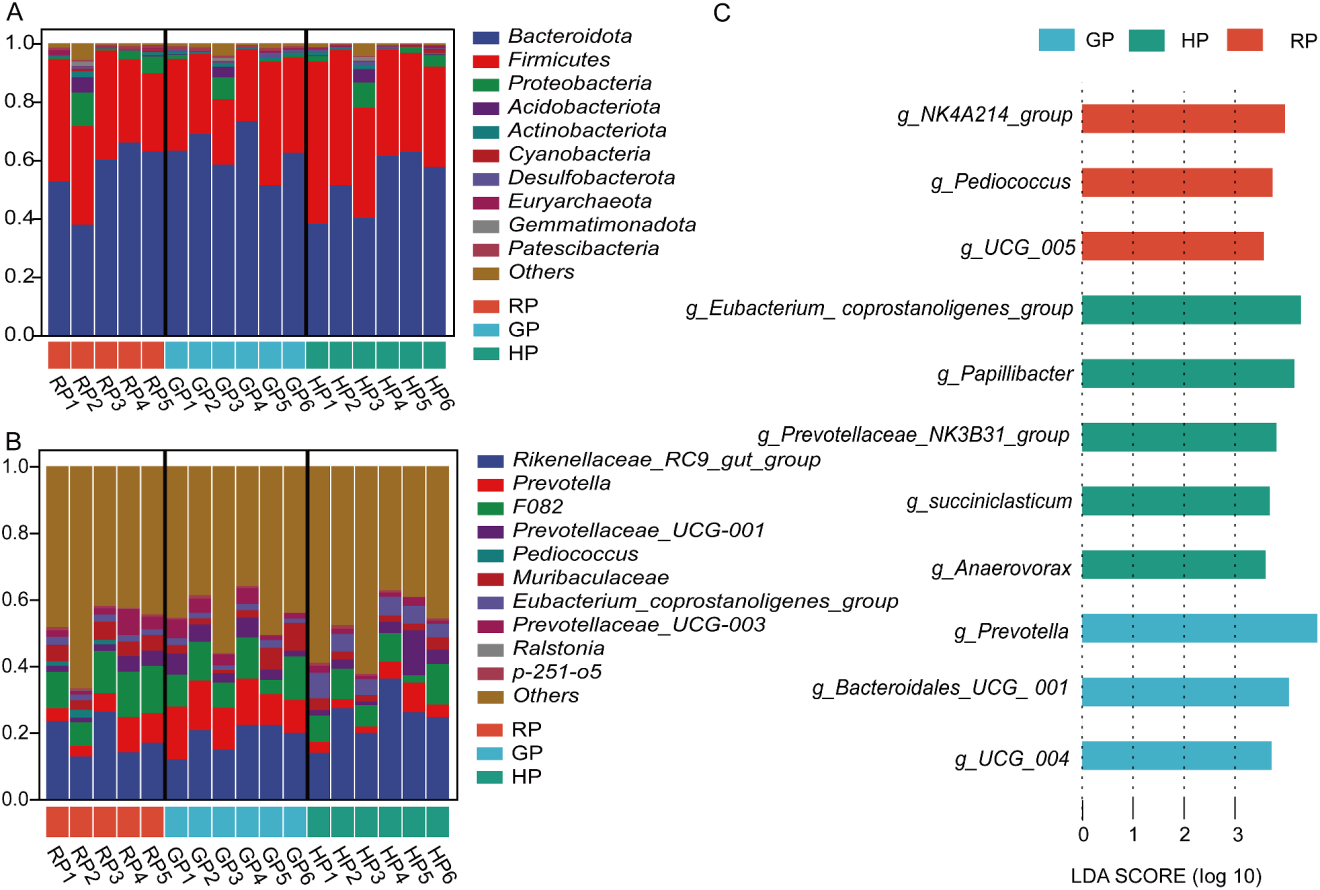
Bacterial composition in the rumen at the phylum (**A**) and genus (**B**) levels. Histogram of the line discriminant analysis (LDA) effect among the three groups (**C**), and the LDA score (log10) > 3.5 were shown

Effects of the alpine meadow in different phenological periods on the bacteria composition at the phylum are presented in Fig. 2A and Table S1. The abundance of Firmicutes was higher in the RP and HP groups than in the GP group *(P* = 0.076). In addition, Patescibacteria abundance was lower in the HP group compared to the RP and GP groups (*P* < 0.001). In addition, there were no differences in the other individual bacteria between the experimental treatments (*P* > 0.05).

At the genus level (Fig. 2B, Table S2), the GP group had higher abundance of *Prevotella* than those in the RP and GP groups (*P* < 0.001). The abundance of *Eubacterium_coprostanoligenes_group* was higher in the HP group compared with the RP and GP groups (*P* < 0.001). Compared with the GP and HP groups, the RP group had higher abundance of *Ralstonia* (*P* = 0.031). The results of the LDA effect size (Fig. 2C) showed that 11 biomarkers were identified as discriminative features between the samples taken from experimental treatments. *NK4A214_group*, *Pediococcus*, and *UCG_005* were enriched as biomarkers in the RP group. Five biomarkers were enriched in the HP group, including *Eubacterium_coprostanoligenes_group*, *Papillibacter*, *Prevotellaceae_NK3B31_group*, *Succiniclasticum*, and *Anaerovorax*. *Prevotella*, *Bacteroidales_UCG_001*, and *UCG_004* were enriched as bacterial biomarkers in the GP group.

### Bacterial composition in the gut

**Fig. 3:**
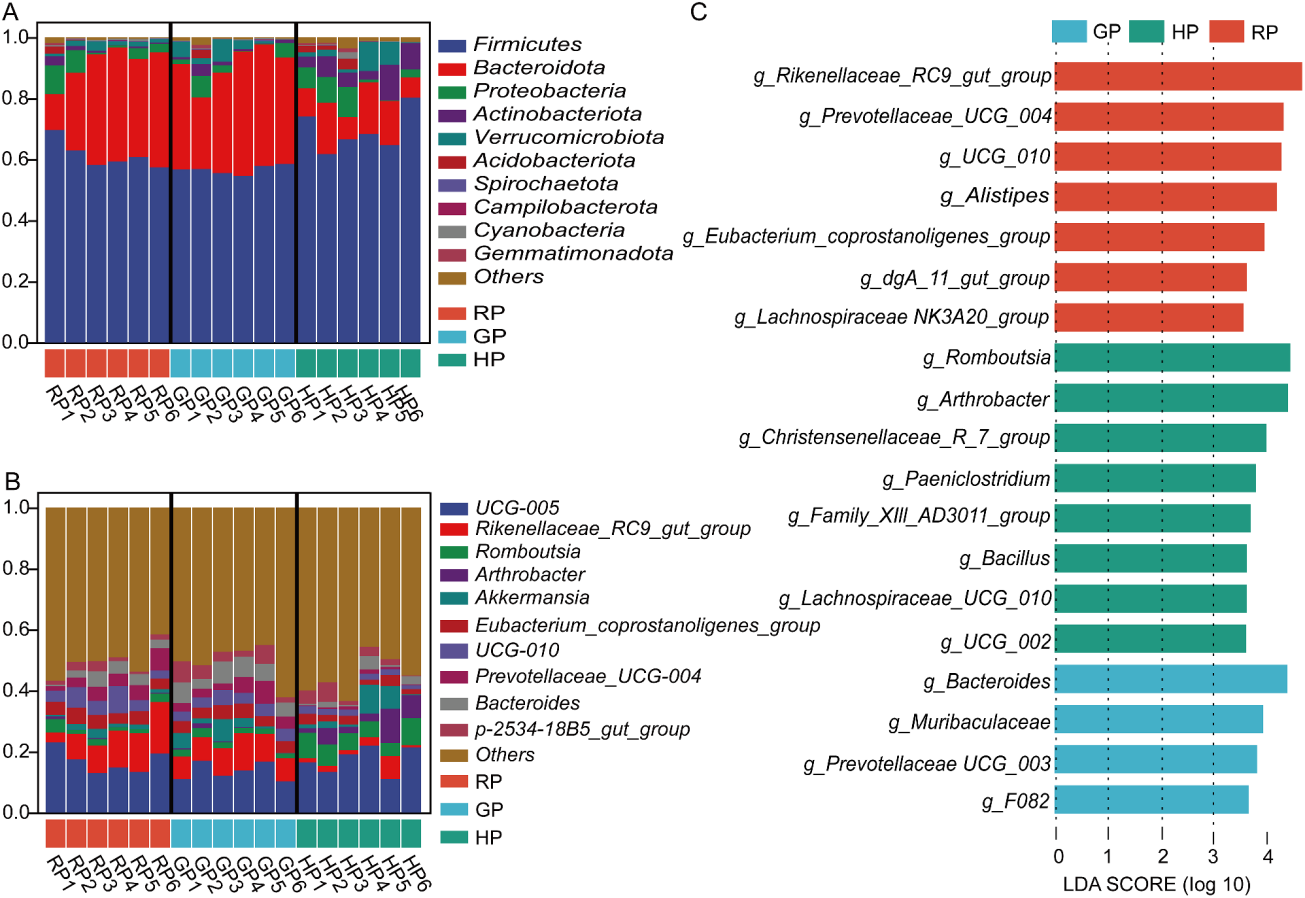
Bacterial composition in the gut at the phylum (**A**) and genus (**B**) levels. The LDA analysis among experimental treatments (**C**), and the LDA score (log10) > 3.5 were shown

The effects of the alpine meadow in different phenological periods on bacterial composition in the gut at the phylum are described in Fig. 3A and Table S3. The abundances of Firmicutes and Actinobacteriota were greater in the HP group than in the RP and GP groups (*P* < 0.01). However, the abundance of Bacteroidota was higher in the RP and GP groups than in the HP group (*P* < 0.001). No differences in the abundances of Proteobacteria, Verrucomicrobiota, and Acidobacteriota were found among the treatments (*P* > 0.05).

As shown in Fig. 3B and Table S4, the abundances of *Rikenellaceae_RC9_gut_group*, *Eubacterium_coprostanoligenes_group*, *UCG-010*, and *Prevotellaceae_UCG-004* were greater in the RP and GP groups than in the HP group (*P* < 0.05). The HP group had higher abundances of *Romboutsia* and *Arthrobacter* than other groups (*P* < 0.01). The abundance of *Bacteroides* was higher in the GP group compared to the RP and HP groups (*P* = 0.002).

The results of the LDA effect size described those 19 biomarkers were found among the three groups (Fig. 3C). Seven biomarkers were enriched in the RP group, including *Rikenellaceae_RC9_gut_group*, *Prevotellaceae_UCG_004*, *UCG_010*, *Alistipesg*, *Eubacterium_coprostanoligenes_group*, *dgA_11_gut_group*, and *Lachnospiraceae NK3A20_group*. Eight biomarkers were enriched in the RP group, including *Romboutsia*, *Arthrobacter*, *Christensenellaceae_R_7_group*, *Paeniclostridium*, *Family_XIAD3011_group*, *Bacillus*, *Lachnospiraceae_UCG_010*, and *UCG-002*. Four biomarkers were enriched in the GP group, including *Bacteroides*, *Muribaculaceae*, *Prevotellaceae_UCG_003*, and *F082*.

### Tax4Fun-based functional predictions in the rumen

**Fig. 4:**
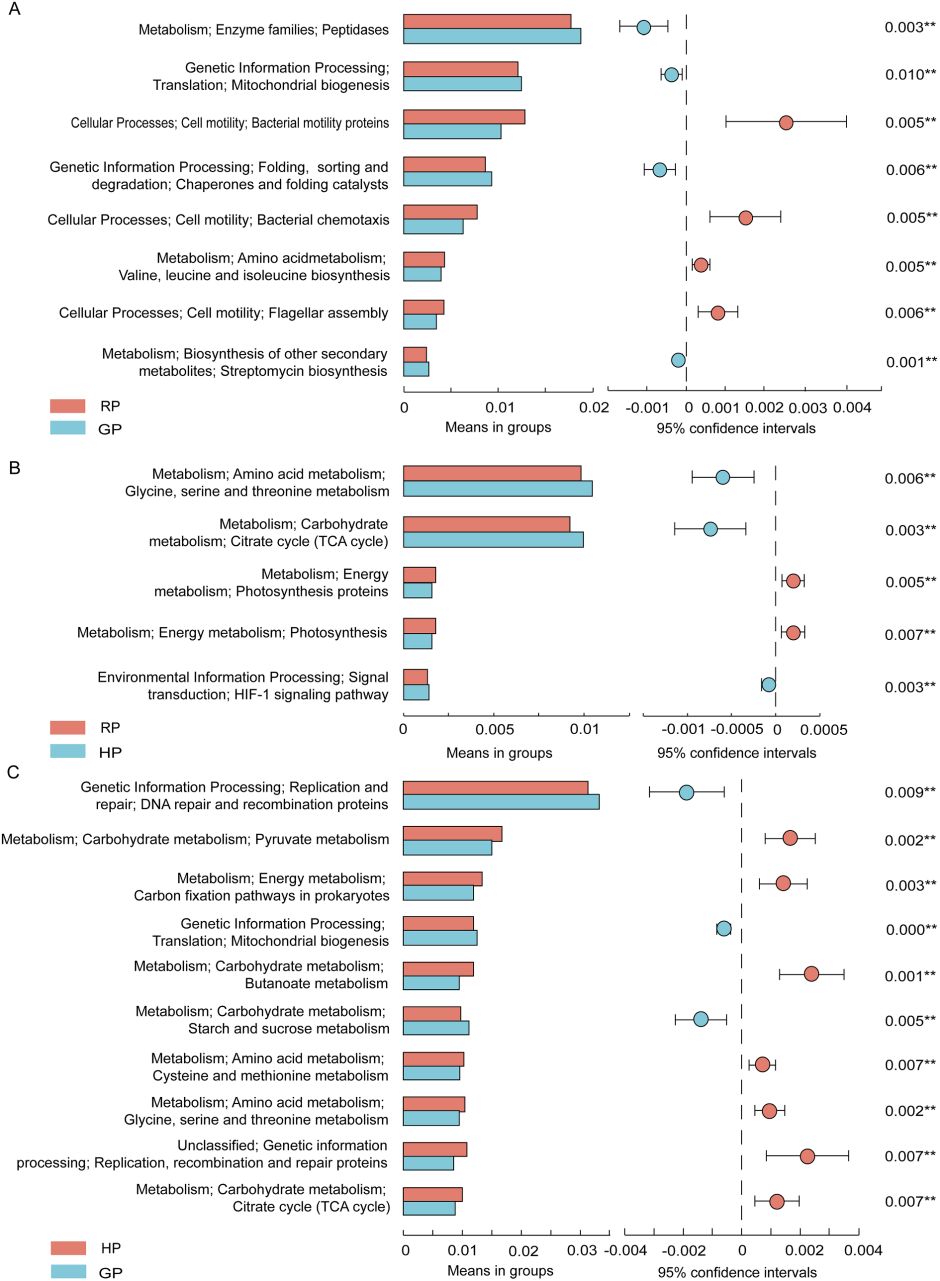
Prediction of bacterial function in the rumen by KEGG between RP and GP groups (**A**), between RP and HP groups (**B**), and between GP and HP groups (**C**) using the Tax4Fun method

Functional predictions based on Tax4Fun-based reveal the important function of rumen microbiome. Compared with the RP group, the metabolism of peptidases, mitochondrial biogenesis, chaperones and folding catalysis, and streptomycin biosynthesis were predicted to get upregulated in the GP group, while the metabolism of bacterial motility proteins, bacterial chemotaxis, valine, leucine and isoleucine biosynthesis, and flagella assembly were predicted to get downregulated (Fig. 4A). The metabolism of glycine, serine and threonine, citrate cycle, and HIF-1 signaling pathway were predicted to get upregulated in the HP group compared with the GP group (Fig. 4B), while the metabolism of photosynthesis proteins and photosynthesis were predicted to get downregulated. Compared to the HP group (Fig. 4C), the metabolism of DNA repair and recombination proteins, mitochondrial biogenesis, and starch and sucrose were predicted to get upregulated in the GP group, whereas the metabolism of pyruvate, carbon fixation pathways in prokaryotes, butanoate, cysteine and methionine, glycine, serine, and threonine were predicted to get downregulated.

### Tax4Fun-based functional predictions in the gut

**Fig. 5:**
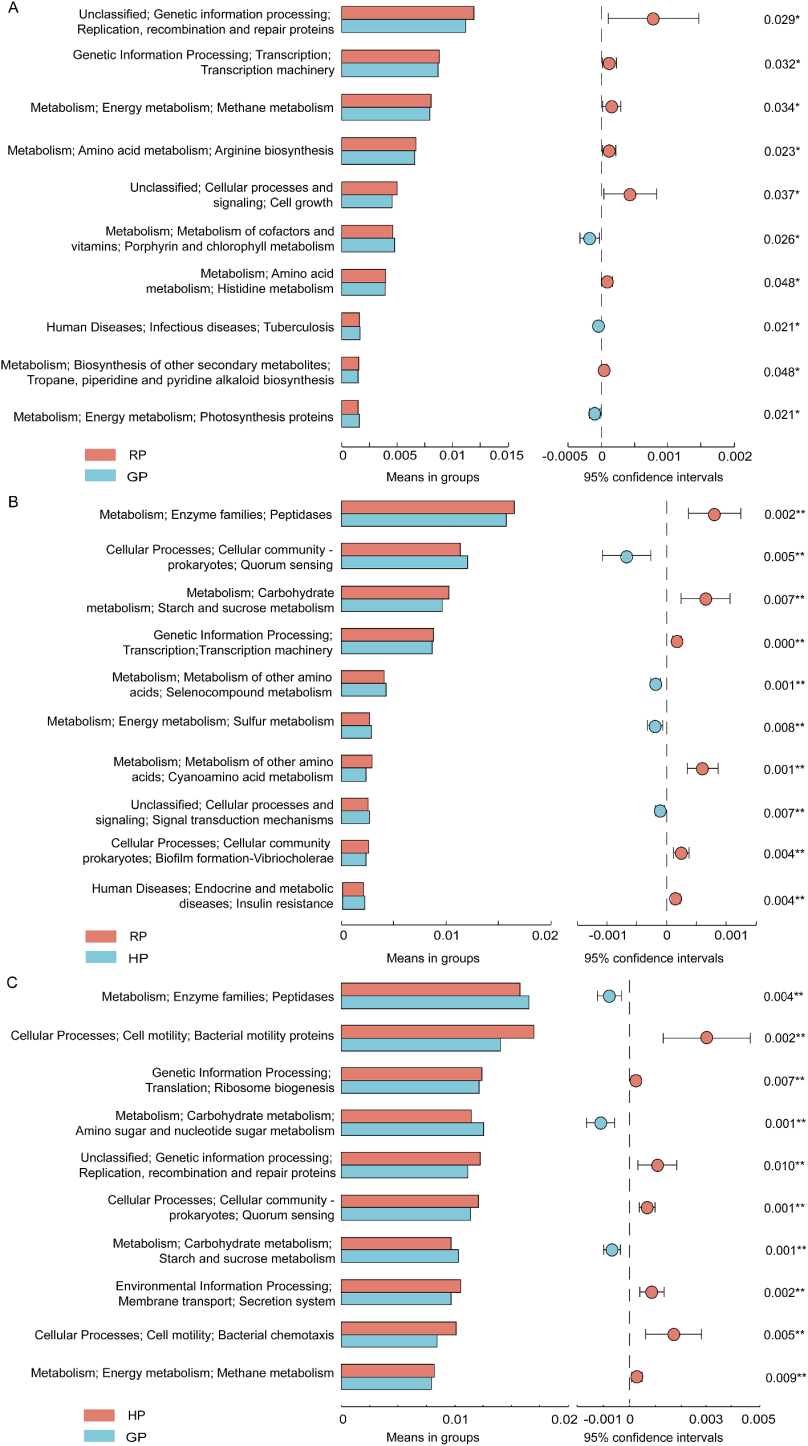
Prediction of bacterial function in the gut by KEGG between RP and GP groups (**A**), between RP and HP groups (**B**), and between GP and HP groups (**C**) by the Tax4Fun method

For the functional predictions in the gut bacteria, the metabolism of replication, recombination and repair proteins, transcription machinery, methane, arginine biosynthesis, and cell growth were predicted to get downregulated in the GP group (Fig. 5A). Compared with the RP group, the metabolism of quorum sensing, selenocompound, and sulfur were predicted to get upregulated in the HP group (Fig. 5B), while the metabolism of peptidases, starch and sucrose, and transcription machinery were predicted to get downregulated. The metabolism of peptidases, amino sugar and nucleotide sugar, and starch and sucrose were predicted to get upregulated in the GP group compared with the HP group, while the metabolism of bacterial motility proteins, ribosome biogenesis, replication, recombination and repair proteins, quorum sensing, and secretion system were predicted to get downregulated (Fig. 5C).

## Discussion

### Bacterial diversity in the rumen and gut

In the present study, a total of 9651 ASVs in the rumen were obtained from samples, and 1611, 2038, and 1771 specific ASVs were observed in the RP, GP, and HP groups, respectively. Higher abundance of ASVs in the GP group was associated with increased levels of bacterial proliferation and growth in the rumen. The alpha diversity indices were similar for all treatments in this study. Combined with the results of rumen bacteria at the phylum, this suggests that the rumen flora maintain a steady state to adapt the nutrient changes on the plateau. In addition, The PERMANOVA test based on the Bray-Curtis distance measures showed that significant differences in bacterial community composition among the treatments. Host-specific bacterial community was altered, which may play an important role in seasonal variations.

In this study, a total of 8540 ASVs were observed in the gut bacterial samples. Among them, 1961, 1561, and 2212 specific ASVs were identified in the RP, GP, and HP groups, respectively, which was not consistent with the results for the rumen bacteria. The higher abundance of ASVs in the HP group suggests that microbes may help the host to use its diet and energy more efficiently, allowing the host to cope better with low-quality forage during the long, cold season. With regard to the alpha diversity, apart from the indices of Chao1 and ASVs, the indices of Simpson and Shannon were greater in the GP group. The PERMANOVA test based on the Bray-Curtis distance measures revealed that significant differences in gut bacterial community composition among three groups. The above findings indicates that the alpine meadow determine the bacterial composition of the gut microbiome.

### Rumen fermentation, bacteria composition in the gastrointestinal tract

In the rumen, the dominated phyla in the grazing yak were Bacteroidetes, followed by Firmicutes in this study. Bacteroidetes and Firmicutes make up 89 − 93% of the total microbiome in all three groups. The distribution of major phyla among microbes closely was consistent with other study. Huang et al. reported that rumen microbes in yaks were dominated by Bacteroidetes (72.13-78.54%) and Firmicutes with the seasonal variation on the Qinghai-Tibetan Plateau [4]. Furthermore, the abundance of Firmicutes was higher in the RP and HP groups, but the ratio of Firmicutes to Bacteroidetes was similar in our study. The ratio of Firmicutes to Bacteroidetes had a positive correlation with feed efficiency and milk-fat yield [7]. In addition, Firmicutes or Bacteroidetes is attributed to the diet, specie, and climate. Within a limited geographical area, diet composition and host species had little effect on the dominant position of these two phyla [8, 9]. Patescibacteria play a role in the genetic information processing, which includes nucleotide metabolic processes, DNA polymerase complex, DNA-templated transcription and translation [10]. The predicted function of rumen bacteria was also found that the metabolism of peptidases, mitochondrial biogenesis, chaperones and folding catalysis were upregulated in the GP group. The higher abundance of Patescibacteria in the RP and GP groups, indicating that the activity of DNA repair was enhanced with the alpine meadow regreen and grassy.

In this study, the GP group had greater abundance of *Prevotella* compared to the RP and HP groups. *Prevotella* (Bacteroidota) involved in hemicellulose, protein metabolism, and starch degradation [11]. *Prevotella* abundance was significantly increased when the diet was switched from low grain to high grain in cows [12]. The metabolism of starch and sucrose was predicted to get upregulated in the GP group. Thus, the higher abundance of *Prevotella* and starch degradation increased the ratio of acetate to propionate, indicating a positive correlation with acetate [13]. Consequently, the cultivation of *Prevotella* and fiber-degrading bacteria can increase the breakdown of plant hemicelluloses (pectin and xylan) and lead to better degradation of fiber [14], which explained the results of acetate and propionate in our study. *Eubacterium* consists of Gram-positive, homogeneous or pleomorphic non-spore forming, obligate anaerobic, chemotrophic bacterial rods that play a key role in energy homeostasis and the suppression of inflammation in the gut [15]. The *Eubacterium_coprostanoligenes_group* (Firmicutes) was higher in the RP group, which demonstrates that yak increases the abortion of energy when alpine meadow regreens.

With regard to the ruminal bacteria, three biomarkers were enriched in the RP group, including *NK4A214_group*, *Pediococcus*, and *UCG_005*. The *NK4A214_group* is member of the *Ruminococcus* family, and these changes may be associated to digest resistant starch. *Pediococcus* belongs to the family *Lactobacillaceae*, and is also considered a safe lactic acid bacterium. It plays an important function in preserving various facets of human fact, such as the nutrient digestion and absorption, resistance against pathogens, and immune system [16], that indicating yak gains more nutrients and energy with the alpine meadow regreen. Three genera were enriched as microbial biomarkers in the GP group, including *Prevotella*, *Bacteroidales_UCG_001*, and *UCG_004*. A previous study showed that *Prevotella* and *Bacteroides* serve as dietary and lifestyle indicators [17]. The genus *Prevotella* is generally observed with high abundance and plays an important part in many processes such as polysaccharide and protein breakdown as well as sugar fermentation [18]. *Bacteroidales* are postulated to be capable of cellulose degradation, and their genomes contain a wide range of plant polysaccharide degradation capabilities [19], which could relate to starch and fiber digestion. In addition, we found five biomarkers were enriched in the HP group, including *Eubacterium_coprostanoligenes_group*, *Papillibacter*, *Prevotellaceae_NK3B31_group*, *Succiniclasticum*, and *Anaerovorax*. *Anaerovorax*, an extreme anaerobic, was found to maintain the stability of the microbial system [20]. *Papillibacter*, *Eubacterium_coprostanoligenes_group*, and *Prevotellaceae_NK3B31_group* are known butyrate producers [21–23], which could not explain the decrease level of butyrate due to low fermentative substrates in the current study. Isobutyrate, isovalerate, and valerate in the rumen were associated with the breakdown of protein and branched-chain amino acids [24]. They are also thought to be stimulating factors that enhance the growth of cellulolytic bacteria. The HP group had higher proportions of isobutyrate, isovalerate, and valerate, which also enhanced the growth of cellulolytic bacteria and fiber degradation.

Microbiome function prediction indicated that most of the metabolic pathways in the rumen were associated with the metabolism of carbohydrates that governed the distribution of carbon, genetic information processing, as well as sugars synthesis and transformation. The metabolism of bacterial motility proteins, bacterial chemotaxis, valine, leucine and isoleucine biosynthesis, and flagella assembly were all upregulated in the RP group. More evidence for a strong correlation between membrane processes, nutrient acquisition, and motility regulation comes from the carbohydrate uptake systems at the bacterial membrane [25]. However, understanding the interactions between bacterial membranes and regulatory functionality in their structure, the possible modifications are still unknown.

### Bacterial composition in the gut

In terms of gut bacteria, the abundance of Firmicutes and the ratio of Firmicutes to Bacteroidota were higher in the HP group, while the abundance of Bacteroidota was higher in the GP group. Firmicutes are usually more prevalent in forage-based diets, while Bacteroidetes are typically more abundant in diets consisting of concentrate [26]. The increase in Bacteroidota was related to the content of starch in the gut. Besides, we found that the abundance of Actinobacteriota was also greater in the HP group. Actinobacteria are extremely versatile producers of bioactive natural products, producing two-thirds of all known antibiotics currently used in the medical today [27]. In accordance with the results obtained, we demonstrated that the bacteria in the gut enhanced fiber utilization in the harsh cold season when yak intakes withered alpine meadow.

At the genus level, the abundances of *Rikenellaceae_RC9_gut_group*, *Eubacterium_coprostanoligenes_group*, and *Prevotellaceae_UCG-004* were greater in the RP and GP groups than in the HP group, which were also as microbe biomarkers. *Rikenellaceae_RC9_gut_group* belongs to the *Rikenellaceae* family from fecal samples and digestive tracts of a wide range of animals, producing propionate, acetate or succinate [28]. *Eubacterium_coprostanoligenes_group* produces mixtures of organic acids from carbohydrates, including the amounts of butyrate and acetate [29]. *Prevotellaceae_UCG_004* can ferment carbohydrates to produce short-chain fatty acids, specifically acetate and butyrate [30]. However, propionate plays a key role in gluconeogenesis and acetate, like butyrate, is lipogenic, suggesting that microbes improve the degradation of polysaccharide in the gut [31]. At present, these genera contained *Bacteroides*, *Muribaculaceae*, *Prevotellaceae_UCG_003*, and *F082* as biomarkers in the GP group. According to the KEGG pathway analysis, these findings were again demonstrated by the remarkable upregulation of peptidases, amino sugar and nucleotide sugar metabolism in the gut bacteria.

In the present study, we found that eight biomarkers of gut bacteria were enriched in the HP group, which contained *Romboutsia*, *Arthrobacter*, *Christensenellaceae_R_7_group*, *Paeniclostridium*, *Family_XIAD3011_group*, *Bacillus*, and *Lachnospiraceae_UCG010*. Yak in the HP group had higher abundances of *Romboutsia* and *Arthrobacter* compared to other groups. *Romboutsia* (Firmicutes) is an obligate anaerobe in the intestine, and its abundance increases in various diseases, such as neurodevelopmental disorders [32]. Furthermore, *Romboutsia* was positively correlated with circulating inflammatory and anxiety-like behaviour [33]. The genus *Arthrobacter* (Actinobacteria), a member of the family *Micrococcaceae*, was confirmed in various extreme, including permanently cold, environments [34]. It is possible that the presence of yak in the harsh cold season on the plateau increases the abundance of *Romboutsia* and *Arthrobacter*, resulting in the microbe disorders and the risk of inflammation. These findings were consistent with the function prediction results in the HP group. The metabolism of bacterial motility proteins, ribosome biogenesis, replication, recombination and repair proteins, quorum sensing, and secretion system were predicted to get upregulated in this study. With respect to metabolic pathways, the metabolism of replication, recombination and repair proteins, transcription machinery, arginine biosynthesis, and cell growth were predicted to get upregulated in the RP group. These findings indicates that bacterial activity and cell growth were associated with the alpine meadow regrowth.

### Serum biochemical indices

Serum biochemical indices serve as a diagnostic marker, revealing the magnitude of lipid mobilization, energy balance, and pathophysiological processes [35]. In the current study, there were no significance differences found in the ALT and AST concentration. These results fall within the typical range of 78 to 132 U/L reported for healthy cattle [36]. The concentrations of these enzymes suggested that the metabolic disorders were not occurred in grazing yak. The CREA is a non-protein nitrogenous compound that is produced by the breakdown of creatine in muscle, which is excreted by glomerular filtration at a constant rate and in the same concentration as in plasma. The CREA is a more dependable measure of kidney function compared to the BUN because it is less affected by external factors like diet and hydration [37]. The BUN, a byproduct of protein breakdown created in the liver and excreted by the kidney, which is often used in combination with CREA to evaluate renal function in clinical [38]. However, it is important to note that BUN content can be influenced by various factors such as protein intake, corticosteroids, gastrointestinal bleeding, and dehydration [39]. The CREA concentration was greater in the HP group (195.46 µmol/L) than in the RP (95.18 µmol/L) and HP groups (166.77 µmol/L) in the present study, which suggests that the higher portion of CREA means the increasing risks of kidney inflammation. Serum biochemicals associated with protein metabolism exhibited a greater variation, and our study revealed that the concentrations of TBA, UREA, and BUN were higher in the RP group. Urea is produced as a result of protein and amino acid metabolism, and the content of ammonia in the rumen is positively correlated with the BUN, reflecting the efficiency of nitrogen metabolism in ruminants [40]. One possible reason for the higher concentrations of UREA and BUN was that the yak increases the its N metabolism with the alpine meadow regreen and grassy. In the present study, the GP group showed higher concentrations of GLU, TG, CHO, HDL, and LDL, indicating the increase of lipid mobilization and energy absorption.

**Fig. 6:**
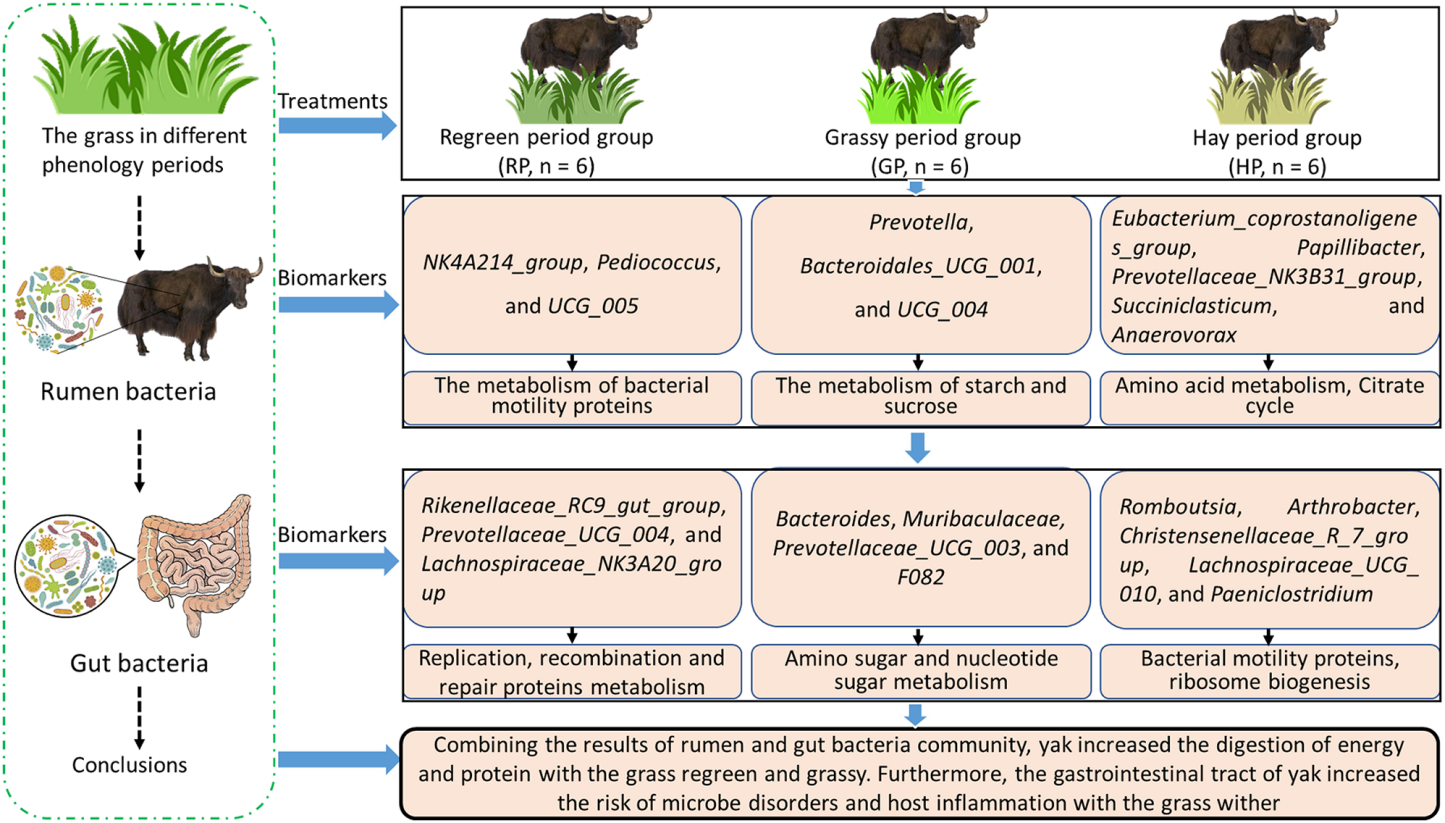
Effects of the alpine meadow in different phenological periods on rumen and gut bacteria community of yak

Taken together, integrating rumen and gut bacteria community, we uncovered some new features of bacterial composition associated with nutrient digestion and host health (Fig. 6). These findings will contribute to changing the grazing pattern according to the different phenological periods.

## Conclusions

We investigated the effects of alpine meadow in different phenological periods on gastrointestinal tract microbes in the grazing yak on the Qinghai-Tibetan Plateau. The RP group exhibited higher concentrations of butyrate, TBA, UBEA, and BUN compared with the GP and HP groups. The concentration of TVFA, acetate, GLU, TG, CHO, HDL, and LDL were higher in the GP group than in the HP. However, compared with the RP and GP groups, the HP group had higher concentrations of isobutyrate, isovalerate, valerate, and CREA. The abundance of *Prevotella* in the rumen, and the abundances of *Rikenellaceae_RC9_gut_group*, *Eubacterium_coprostanoligenes_group*, and *Prevotellaceae_UCG-004* in the gut were higher in the GP group compared with the HP group. Compared with the RP and GP groups, the HP had higher abundance of *Eubacterium_coprostanoligenes_group* in the rumen as well as the abundances of *Romboutsia* and *Arthrobacter* in the gut. In summary, the carbohydrates digestion of grazing yak would be higher with the alpine meadow regreen and grassy due to the gastrointestinal tract microbes. However, the risk of microbe disorders and host inflammation in grazing yak were higher with the alpine meadow wither. These findings have potential to play an important function in understanding the role of microbes in the grazing yak with alpine meadow growth, which also provides an experimental basis for the grazing patterns.

## Methods

### Experimental design and management

The experiment was conducted at Haibei Demonstration Zone of Plateau Modern Ecological Animal Husbandry Science and Technology (E100°95′, N36°91′, about the altitude of 3100 m), Haiyan County, Qinghai Province, China. The mean annual air temperatures of 1.5 °C, and a mean annual precipitation of 400 mm. The vegetation consisted of typical alpine meadows. At the study sites, yaks commonly grazed in a full-grazing system with alpine meadow as the only feed. The dominant species were *Kobresia pygmaea*, *Kobresia humilis*, *Elymus dahuricus*, and *Poa annua*.

A total of eighteen 3-year-old female yaks with a similar body weight of 130 ± 19 kg were randomly divided into three groups, with six yaks per group. Based on the plant phenological period of the Qinghai-Tibetan Plateau, yaks were assigned to the following three treatments: (1) regreen period group (RP, *n* = 6); (2) grassy period group (GP, *n* = 6); and (3) hay period group (HP, *n* = 6). All yaks had free access to fresh water and grazed in the same grassland. At the end of the relevant experimental periods, samples were collected at the regreen period (10 June), grassy period (10 September), and hay period (10 December), respectively.

A total of 18 randomly selected quadrats (50 cm × 50 cm) were chosen from the grassland on which the yaks grazed. Mixed alpine meadow samples were collected and edible grass was retained. Then, the alpine meadow samples were dried at 65 °C for 48 h, then passed through a 1-mm screen. The content of DM, organic matter (OM), and CP in alpine meadow were evaluated according to the procedures of AOAC [41]. The NDF and ADF were measured using the fiber analyzer (A2000, Ankom Technology, Fairport, NY) following the methods of Van Soest et al. [42]. The nutrients and chemical composition in the alpine meadow (%, DM basis) are presented in Table S5.

Blood samples were collected in the day before slaughter from the jugular vein of each yak into serum tubes, and then placed a centrifuge 3000 g for 10 min at 4 °C. After centrifugation, the serum samples were separated, transferred into 5 mL vials, and frozen at -80 °C until further analysis.

The experiment was conducted in accordance with the guidelines of Institution of Animal Care and Ethics Committee of the Northwest Institute of Plateau Biology, Chinese Academy of Sciences, and animal welfare and conditions were considered in the use of experimental animals. All yaks were transported to a nearby commercial slaughterhouse (Xiahua Halal Meat Food Co., Ltd., Qinghai Province, China) and fasted a 12 h from solids and liquids. The next morning, before the Halal slaughter process, each yak was stunned with a pneumatic hammer. Then, one samples of rumen fluid (5 mL) was preserved at -20 °C for determination of VFAs, and the other samples (5 mL) were collected and stored in liquid nitrogen until analysis of the ruminal bacteria. The rectal contents were collected and stored immediately in liquid nitrogen, and transported to the laboratory frozen at -80 °C until assayed. Unfortunately, one sample of rumen fluids in the RP group was missing.

### Sample analysis

The thawed samples of rumen fluid were centrifuged at 10,000 r/min at 4 °C for 10 min, and the supernatants were determined following the method of Liang et al. [43]. The concentrations of VFAs were determined by gas chromatography (GC) on an Agilent7890A (Agilent, CA, USA) equipped with a flame-ionization detector (FID). The GC was installed with a DB-WAX column (30 m × 0.25 μm × 0.25 μm, Agilent Technologies Co., Ltd, Santa Clara, CA, USA). The injector temperature was set to 200 °C, and the detector was set to 240 °C. The temperature program was defined as follows: the temperature was raised from 60 to 180 °C at a speed of 10 °C/min and maintained for 5 min, then, the temperature was raised to 250 °C at a rate of 20 °C /min for 5 min, and was held for 5 min.

### Serum biochemical analysis

The ALT, glutamic oxaloacetic transaminase (AST), albumin (ALB), total bile acids (TBA), BUN, CREA, GLU, CHO, HDL, LDL, and TG were measured using an automatic biochemical analyzer (Chemray-800; Rayto Life and Analytical Sciences Co., Ltd. Shenzhen, China).

### Extraction of bacterial DNA

Bacterial DNA was extracted using the CTAB/SDS method. The concentration and purity of DNA were detected using 1% agarose gels and diluted to a concentration of 1 ng/µL with sterile water. The specific PCR primers of 338F (5’-ACTCCTACGGGAGGCAGCAG3-3’) and 806R (5’-GGACTACHVGGGTWTCTAAT-3’) were used to amplify the V3-V4 region of the 16 S rRNA gene. The PCR reactions contained 15 µL of Phusion® High-Fidelity PCR Master Mix (New England Biolabs), 0.2 µM of forward and reverse primer, and 10 ng of target DNA. The PCR incubation conditions included the initial denaturation step at 98 °C for 1 min, followed by 30 cycles at 98 °C for 10 s, 50 °C for 30 s, and 72 °C for 30 s, and finally a 5 min extension at 72 °C. PCR products were analyzed by 2% agarose gel electrophoresis, and recovered with Qiagen Gel Extraction Kit (Qiagen, German). The sequencing library was created by the NEBNext® Ultra™ IIDNA Library Prep Kit (Cat No. E7645) following the manufacturer instructions. The quality of library was evaluated by the Qubit@ 2.0 Fluorometer (Thermo Scientific) and Agilent Bioanalyzer 2100 system. Then, sequencing was performed using the Illumina NovaSeq platform producing 250 bp paired-end reads.

The paired-end reads of DNA segments were merged using the FLASH software. The amplicon sequences were performed using the QIIME package. The initial amplicon sequence variants (ASVs) were generated using the denoise function of DADA2. Quality filtering was performed using the abundances of ASVs smaller than 5. The abundances of ASVs were normalized by the number of sequences corresponding to the sample with the smallest number of sequences. The absolute abundances of ASVs were also standardized using a reference sequences number corresponding to the sample with the fewest sequences. The normalized data was then used for subsequent alpha diversity and beta diversity analyses.

The LEfSe software (version 1.0) was used to perform LEfSe analysis (line discriminant analysis (LDA) score threshold: 3.5) to identify the biomarkers. All raw sequences were deposited in the NCBI Sequence Read Archive (SRA) database and can be accessed via accession number: PRJNA99034.

### Statistical analysis

Alpha diversity including ASVs, Chao1, Shannon, and Simpson was calculated using the software of QIIME2. Beta diversity was determined by QIIME2 using the Bray-Curtis distance. Permutational multivariate analysis of variance (PERMANOVA) was performed with R-package “vegan” to test the differences among the groups (version 4.1.3). The rumen fermentation paraments, serum biochemical indices, and relative abundance of bacteria taxa in the rumen and gut was conducted using SPSS statistical software (version 24.0, IBM, Armonk, NY, United States). The significance difference between the treatments was examined using one-way ANOVA. The statistical model was as follows: Yi = µ + Ai + Bi, in which Yi, µ, Ai, and Bi represented the dependent variable, overall mean, treatment effect, and error term, respectively. Duncan’s test was carried out for multiple comparisons of treatment means when a *P* ≤ 0.05 indicating significance, and a tendency for a treatment effect was detected when 0.05 < *P* ≤ 0.10.

### Electronic supplementary material

Below is the link to the electronic supplementary material.

**Supplementary Material 1: Table S1.** Effects of the alpine meadow in different phenological periods on the rumen bacteria at the phylum level (%). **Table S2.** Effects of the alpine meadow in different phenology periods on the rumen bacteria at the genus level (%). **Table S3.** Effects of the alpine meadow in different phenological periods on the gut bacteria at the phylum level (%). **Table S4.** Effects of the alpine meadow in different phenological periods on the gut bacteria at the genus level (%). **Table S5.** The nutrients and chemical composition in the alpine meadow (%, DM basis)

## Data Availability

The raw sequence data in the study has been deposited in the NCBI database and can be accessed via web links (https://dataview.ncbi.nlm.nih.gov/object/PRJNA990348?reviewer=qmsb9dgptgomn937vhb2b73a9d).

## Abbreviations

ADF: acid detergent fiber
ALB: albumin
ALT: glutamic-pyruvic transaminase
AST: glutamic oxaloacetic transaminase
ASVs: amplicon sequence variants
BUN: blood urea nitrogen
CHO: cholesterol
CP: crude protein
CREA: creatinine
DM: dry matter
GLU: glucose
GP: grassy period
HDL: high density lipoprotein
HP: hay period
LDA: line discriminant analysis
LDL: low density lipoprotein
NDF: neutral detergent fiber
OM: organic matter
PCoA: principal coordinate analysis
RP: regreen period
TBA: total bile acids
TG: triglyceride
UREA: urea
VFA: volatile fatty acids
