# Supplementary material for: Effects of the alpine meadow in different phenological periods on rumen fermentation and gastrointestinal tract bacteria community in grazing yak on the Qinghai-Tibetan Plateau
Source: BMC Microbiol. 2024 Feb 19;24:62. doi: 10.1186/s12866-024-03182-y (PMC10875897; doi:10.1186/s12866-024-03182-y)
Supplement: Supplementary file 1 — Supplementary Material 1: Table S1. Effects of the alpine meadow in different phenological periods on the rumen bacteria at the phylum level (%). Table S2. Effects of the alpine meadow in different phenology periods on the rumen bacteria at the genus level (%). Table S3. Effects of the alpine meadow in different phenological periods on the gut bacteria at the phylum level (%). Table S4. Effects of the alpine meadow in different phenological periods on the gut bacteria at the genus level (%). Table S5. The nutrients and chemical composition in the alpine meadow (%, DM basis) [file 12866_2024_3182_MOESM1_ESM.docx]

**Supplementary Table S1.** Effects of the alpine meadow in different phenological periods on the rumen bacteria at the phylum level (%).

|  | RP | GP | HP | SEM^1^ | *P*-value |
| --- | --- | --- | --- | --- | --- |
| Bacteroidota | 0.559 | 0.630 | 0.520 | 0.025 | 0.186 |
| Firmicutes | 0.337^a^ | 0.302^b^ | 0.408^a^ | 0.020 | 0.076 |
| Proteobacteria | 0.041 | 0.017 | 0.028 | 0.008 | 0.559 |
| Acidobacteriota | 0.011 | 0.006 | 0.008 | 0.004 | 0.901 |
| Actinobacteriota | 0.009 | 0.008 | 0.006 | 0.001 | 0.728 |
| Cyanobacteria | 0.004 | 0.001 | 0.003 | 0.001 | 0.282 |
| Desulfobacterota | 0.003 | 0.007 | 0.006 | 0.001 | 0.381 |
| Euryarchaeota | 0.005 | 0.003 | 0.004 | 0.001 | 0.587 |
| Gemmatimonadota | 0.003 | 0.002 | 0.002 | 0.001 | 0.918 |
| Patescibacteria | 0.009^a^ | 0.009^a^ | 0.003^b^ | 0.001 | < 0.001 |
| Others | 0.018 | 0.016 | 0.012 | 0.004 | 0.856 |
| Firmicutes/ Bacteroidota | 0.634 | 0.492 | 0.838 | 0.069 | 0.101 |

^1^SEM, standard error of the mean; a − c means within a row with different subscripts differ when *p*-value < 0.05

**Supplementary Table S2.** Effects of the alpine meadow in different phenology periods on the rumen bacteria at the genus level (%).

|  | RP | GP | HP | SEM^1^ | *P*-value |
| --- | --- | --- | --- | --- | --- |
| *Rikenellaceae_RC9_gut_group* | 0.187 | 0.187 | 0.247 | 0.015 | 0.183 |
| *Prevotella* | 0.064^b^ | 0.127^a^ | 0.042^b^ | 0.011 | < 0.001 |
| *F082* | 0.117 | 0.098 | 0.078 | 0.008 | 0.159 |
| *Prevotellaceae_UCG-001* | 0.029 | 0.042 | 0.044 | 0.007 | 0.683 |
| *Pediococcus* | 0.010^a^ | 0.000^b^ | 0.000^b^ | 0.002 | 0.013 |
| *Muribaculaceae* | 0.045 | 0.038 | 0.026 | 0.005 | 0.277 |
| *Eubacterium_coprostanoligenes_group* | 0.020^b^ | 0.018^b^ | 0.055^a^ | 0.005 | < 0.001 |
| *Prevotellaceae_UCG-003* | 0.033 | 0.035 | 0.016 | 0.005 | 0.164 |
| *Ralstonia* | 0.001^a^ | 0.000^b^ | 0.000^b^ | 0.000 | 0.031 |
| *p-251-o5* | 0.006 | 0.005 | 0.007 | 0.001 | 0.667 |
| *Others* | 0.488 | 0.452 | 0.486 | 0.022 | 0.765 |

^1^SEM, standard error of the mean; a − c means within a row with different subscripts differ when *p*-value < 0.05.

**Supplementary Table S3**. Effects of the alpine meadow in different phenological periods on the gut bacteria at the phylum level (%).

|  | RP | GP | HP | SEM^1^ | *P*-value |
| --- | --- | --- | --- | --- | --- |
| Firmicutes | 0.614^b^ | 0.567^b^ | 0.693^a^ | 0.016 | 0.001 |
| Bacteroidota | 0.301^a^ | 0.344^a^ | 0.119^b^ | 0.029 | < 0.001 |
| Proteobacteria | 0.041 | 0.027 | 0.048 | 0.008 | 0.580 |
| Actinobacteriota | 0.010^b^ | 0.012^b^ | 0.063^a^ | 0.008 | 0.001 |
| Verrucomicrobiota | 0.016 | 0.030 | 0.036 | 0.006 | 0.445 |
| Acidobacteriota | 0.004 | 0.005 | 0.012 | 0.003 | 0.454 |
| Spirochaetota | 0.001 | 0.002 | 0.000 | 0.000 | 0.139 |
| Campilobacterota | 0.001 | 0.000 | 0.000 | 0.000 | 0.336 |
| Cyanobacteria | 0.005 | 0.002 | 0.005 | 0.001 | 0.536 |
| Gemmatimonadota | 0.001 | 0.002 | 0.004 | 0.001 | 0.566 |
| Others | 0.006^b^ | 0.009^b^ | 0.019^a^ | 0.002 | 0.039 |
| Firmicutes/ Bacteroidota | 2.504^b^ | 1.704^b^ | 6.909^a^ | 0.739 | 0.002 |

^1^SEM, standard error of the mean; a − c means within a row with different subscripts differ when *p*-value < 0.05.

**Supplementary Table S4.** Effects of the alpine meadow in different phenological periods on the gut bacteria at the genus level (%).

|  | RP | GP | HP | SEM^1^ | *P*-value |
| --- | --- | --- | --- | --- | --- |
| *UCG-005* | 0.168 | 0.135 | 0.172 | 0.009 | 0.205 |
| *Rikenellaceae_RC9_gut_group* | 0.104^a^ | 0.088^a^ | 0.026^b^ | 0.011 | 0.002 |
| *Romboutsia* | 0.022^b^ | 0.020^b^ | 0.066^a^ | 0.006 | < 0.001 |
| *Arthrobacter* | 0.003^b^ | 0.004^b^ | 0.050^a^ | 0.007 | 0.003 |
| *Akkermansia* | 0.015 | 0.030 | 0.036 | 0.007 | 0.454 |
| *Eubacterium_coprostanoligenes_group* | 0.042^a^ | 0.040^a^ | 0.025^b^ | 0.002 | 0.003 |
| *UCG-010* | 0.050^a^ | 0.039^a^ | 0.020^b^ | 0.004 | 0.006 |
| *Prevotellaceae_UCG-004* | 0.042^a^ | 0.041^a^ | 0.007^b^ | 0.005 | 0.003 |
| *Bacteroides* | 0.031^b^ | 0.057^a^ | 0.017^b^ | 0.005 | 0.002 |
| *p-2534-18B5_gut_group* | 0.018 | 0.041 | 0.029 | 0.005 | 0.161 |
| *Others* | 0.504 | 0.506 | 0.552 | 0.014 | 0.322 |

^1^SEM, standard error of the mean; a − c means within a row with different subscripts differ when *p*-value < 0.05.

**Supplementary Table S5.** The nutrients and chemical composition in the alpine meadow (%, DM basis)

| Items | RP | GP | HP |
| --- | --- | --- | --- |
| DM | 94.55 | 94.84 | 97.34 |
| OM | 86.57 | 85.76 | 92.11 |
| NDF | 48.5 | 47.91 | 50.13 |
| ADF | 23.05 | 23.50 | 25.84 |
| CP | 13.80 | 8.76 | 4.60 |
